# Development of a TaqMan-Probe-Based Multiplex Real-Time PCR for the Simultaneous Detection of African Swine Fever Virus, Porcine Circovirus 2, and Pseudorabies Virus in East China from 2020 to 2022

**DOI:** 10.3390/vetsci10020106

**Published:** 2023-02-01

**Authors:** Huaicheng Liu, Jianwen Zou, Rongchao Liu, Jing Chen, Xiaohan Li, Haixue Zheng, Long Li, Bin Zhou

**Affiliations:** 1 MOE Joint International Research Laboratory of Animal Health and Food Safety, College of Veterinary Medicine, Nanjing Agricultural University, Nanjing 210095, China; 2 Lanzhou Veterinary Research Institute, Chinese Academy of Agricultural Sciences, Lanzhou 730000, China; 3 College of Animal Science and Technology, College of Veterinary Medicine, Zhejiang A&F University, Hangzhou 311300, China

**Keywords:** multiplex real-time PCR, African swine fever virus, porcine circovirus 2, pseudorabies virus, detection, mixed infection

## Abstract

**Simple Summary:**

At present, mixed infection with multiple pathogens is an important reason for the complexity of swine diseases in China. Among them, African swine fever virus (ASFV), porcine circovirus 2 (PCV2), and pseudorabies virus (PRV) are extremely important DNA viruses that cause reproductive abnormalities in sows. Therefore, there is an urgent need for rapid and sensitive detection methods that can diagnose three kinds of DNA viruses in the clinic. Herein, a multiplex real-time PCR (qPCR) assay based on TaqMan probes was developed for the simultaneous determination of ASFV, PCV2, and PRV. To ascertain the use of the multiplex real-time qPCR, 383 field specimens from the pig farms of four provinces in East China were collected. The survey data displayed that the ASFV, PCV2, and PRV single infection rates were 22.45%, 28.46%, and 2.87%, respectively. The mixed infection rates of ASFV + PCV2, ASFV + PRV, PCV2 + PRV, and ASFV + PCV2 + PRV were 5.22%, 0.26%, 1.83%, and 0.26%, respectively. The assay established in this study could be used as a differential diagnostic tool for the monitoring and control of ASFV, PCV2, and PRV in the field.

**Abstract:**

African swine fever virus (ASFV), porcine circovirus 2 (PCV2), and pseudorabies virus (PRV) are important DNA viruses that cause reproductive disorders in sows, which result in huge losses in pig husbandry, especially in China. The multiplex qPCR assay could be utilized as a simultaneous diagnostic tool for field-based surveillance and the control of ASFV, PCV2, and PRV. Based on the conserved regions on the p72 gene of ASFV, the Cap gene of PCV2, the gE gene of PRV, and the porcine endogenous β-Actin gene, the appropriate primers and probes for a multiplex TaqMan real-time PCR test effective at concurrently detecting three DNA viruses were developed. The approach demonstrated high specificity and no cross-reactivity with major pathogens related to swine reproductive diseases. In addition, its sensitivity was great, with a detection limit of 10^1^ copies/L of each pathogen, and its repeatability was excellent, with intra- and inter-group variability coefficients of <2%. Applying this assay to detect 383 field specimens collected from 2020 to 2022, the survey data displayed that the ASFV, PCV2, and PRV single infection rates were 22.45%, 28.46%, and 2.87%, respectively. The mixed infection rates of ASFV + PCV2, ASFV + PRV, PCV2 + PRV, and ASFV + PCV2 + PRV were 5.22%, 0.26%, 1.83%, and 0.26%, respectively. Overall, the assay established in this study provides an effective tool for quickly distinguishing the viruses causing sow reproductive disorders, suggesting its huge clinical application value in the diagnosis of swine diseases.

## 1. Introduction

In China, the breeding density and scale of pig herds are increasing. The pigs in some large farms are experiencing mixed infection or secondary infection with multiple pathogens, leading to high morbidity and mortality [1]. Severe pathogens, such as *toxoplasma gondii*, porcine respiratory and reproductive syndrome virus, classical swine fever virus, porcine parvovirus, Japanese encephalitis virus, African swine fever virus (ASFV), porcine circovirus (PCV), and pseudorabies virus (PRV), can cause sow reproductive disorders. At present, among these pathogens, the porcine respiratory and reproductive syndrome virus is the most concerning, but three DNA viruses, ASFV, PCV2, and PRV, also cannot be ignored. At the same time, because PCV2 can inhibit type I interferon induction to promote the infections caused by other DNA viruses [2,3], the problem of mixed infection with ASFV, PCV2, and PRV is more prominent [4,5,6]. African swine fever (ASF) caused by ASFV is an acute, febrile, and highly lethal infectious disease of pigs that requires notifying the World Organization for Animal Health (WOAH) [7]. Since 2018, there have been some outbreaks in mainland China within the scope of the epidemic, seriously endangering the pig-breeding industry in China and causing huge economic losses [8]. ASFV is a large-enveloped, double-stranded DNA virus, and its genome consists of a linear double-stranded DNA molecule of 170–190 kb coding for more than 50 structural proteins and several non-structural proteins. It is well known that ASFV molecular polymorphism has been investigated by partial sequencing of the gene encoding the major capsid protein p72 [9,10,11]. The term porcine circovirus-associated disease (PCVAD) refers to several swine diseases induced by porcine circovirus (PCV). Sow reproductive abnormalities form one of the typical symptoms. The identified PCVs are PCV1, PCV2, PCV3, and PCV4. In recent years, PCV3 and PCV4 have been discovered, but there is no direct evidence that they cause reproductive disorders in sows [12,13]. PCV2 continues to have the greatest clinical impact. PCV2 is a small, circular, single-stranded DNA virus belonging to the family Circoviridae, genus Circoviruse [14]. ORF1 and ORF2 are two major ORFs situated on opposite strands that respectively encode Rep and Cap proteins [15]. PRV is an enveloped, double-stranded DNA herpesvirus belonging to the Varicellovirus genus of the Alphaherpesvirinae subfamily of the Herpesviridae family [16]. PR is an acute infectious disease that can cause fatal infection of piglets and abortion of sows and can infect a variety of domestic and wild animals such as cattle, sheep, dogs, rabbits, mice, and minks [17,18,19,20]. In view of the similarity of the clinical symptoms of ASF, PR, and PCVAD in the early stages of these three diseases, resulting in a difficult diagnosis in the field, it is necessary to develop a differential diagnosis for these three viruses.

These three viruses are difficult to distinguish using clinical symptoms alone. At present, diagnosis mostly depends on molecular diagnosis. Considering the convenience, sensitivity, and rapidity of identifying clinical viral infections in pigs, several technologies, such as PCR, real-time PCR, and digital PCR, are frequently used. There are also some articles about isothermal nucleic acid amplification, such as recombinase polymerase amplification (RPA), recombinase-aided amplification (RAA), and loop-mediated isothermal amplification (LAMP) [21,22,23,24,25,26,27]. Combining CRISPR technology with flow immunochromatography to detect viruses has yielded positive results in some studies [28]. These methods exhibit excellent sensitivity and specificity, but no mature commercial applications exist. In clinical detection, qPCR is the method most frequently employed. However, repeated detection of different pathogens wastes time and makes operations cumbersome, necessitating the development of an assay that can be used to simultaneously detect and discriminate the swine pathogens. Multiplex real-time PCR (qPCR) is a method that can detect multiple pathogens in a single reaction system, which has the advantages of high sensitivity, good accuracy, and direct analysis of results without electrophoresis compared with PCR [29,30,31,32,33]. Additionally, it greatly reduces the cost of materials and time required of the testers in the detection process, especially for large-scale specimen detection and specimen-mixing infection detection. Several single real-time qPCR assays have been published that detect ASFV, PRV, or PCV2. To distinguish between genotype I and genotype II strains of ASFV in the Chinese epidemic strain, a duplex real-time qPCR test has been devised. Multiplex real-time qPCR methods to detect ASFV, classical swine fever virus (CSFV), and atypical porcine pestivirus (APPV) have been developed. To date, no assay has been described for the simultaneous detection of ASFV, PRV, and PCV2. In this investigation, a multiplex real-time qPCR assay was established to simultaneously detect and discriminate ASFV, PCV2, and PRV. More importantly, we applied the established method to detect and analyze field specimens of swine from four provinces in East China and found mixed infections, providing data support for formulating prevention and control measures in this region.

## 2. Materials and Methods

### 2.1. Special Primers and Probes Design

Special primers and probes were designed based on the conserved regions of the ASFV (MK333180.1), PCV2 (KT719404.1), and PRV (KT809429.1) for the creation of constructed plasmid standards and the establishment of a real-time qPCR assay. An internal reference, β-Actin, was amplified as a control [34]. The primers and probes manufactured by Generay Biotechnology Co., Ltd. (Shanghai, China), are listed in Table 1.

### 2.2. Positive DNA/cDNA and Field Specimens

Positive cDNA of JEV and CSFV were from the NJ-2008 strain (GQ918133) and the Shimen strain (AF092448). The positive DNA of PRV was from the SD-2019 strain (GenBank accession No. MW805231). Other positive DNA or cDNA of PCV1, PCV2, PCV3, PCV4, PRRSV, PPV, *Chlamydia suis*, and *Toxoplasma gondii* were from specimens previously confirmed using PCR and RT-PCR assays. ASFV-positive DNA was a gift from the Lanzhou Veterinary Research Institute (Lanzhou, China). All the positive nucleic acid above of swine pathogens was stored in the lab for specificity experiments. In all, 383 field specimens, consisting of lymphoid tissues, placentas, sera, and blood, were collected from sows with reproductive issues and aborted piglets in the pig farms of four provinces of East China from 2020 to 2022. The information of the specimens was shown in Table 2.

### 2.3. Construction of the Recombinant Plasmid

The conserved regions of ASFV p72, PCV2 Cap, or PRV gE were cloned into a pEasy vector and produced three recombinant plasmids, named pEasy-ASFV, pEasy-PCV2, and pEasy-PRV, and sent to Nanjing GenScript Biotechnology Co., Ltd. (Nanjing, China), for DNA sequencing. The concentration of plasmid standards was determined using a NanoDrop spectrophotometer (Thermo Fisher, Waltham, MA, USA). Finally, the copy numbers of the three recombinant plasmids were calculated [35].

### 2.4. Optimization of Reaction Conditions for the Multiplex Real-Time qPCR

The optimal reaction conditions were determined according to a previous report [33]. In brief, the 10-fold diluted plasmids constructed above from 3 × 10^7^ copies/μL to 3 × 10^4^ copies/μL were amplified as the templates. Firstly, a mixture of three diluted plasmids with a concentration of up to 1 × 10^4^ copies/μL was prepared. Secondly, four mixed pairs of customized primers and probes were added to one reaction condition and set to determine the optimal work concentrations for the development of this assay. The final volume of 20 μL included 10 μL 2 × AceQ qPCR Probe Master Mix (Vazyme, Nanjing, China), 0.1–1.8 μL of each of the primers, 0.1–0.8 μL of each of the probes, and 2 μL of templates. Thirdly, FAM, HEX, Texas Red, and Cy5 were configured as the fluorescence channels for the real-time qPCR instrument’s four fluorescence channels. Finally, a commercialized real-time PCR instrument (IDEXX, Westbrook, ME, USA) was used to collect the fluorescence signals.

### 2.5. Establishment of Standard Curves of the Multiplex Real-Time qPCR

According to the report described previously [33], the amplification standard curves were generated. In brief, three constructed plasmid standards of 10-fold serial dilutions (10^7^ to 10^1^ copies/μL) in distilled water were employed as amplification templates for this assay. The logarithm of the copy number determined the horizontal axis of the standard curve, while the vertical axis was determined by the Ct value. The Prism software was used to draw the standard curves.

### 2.6. Specificity, Sensitivity, and Repeatability of the Multiplex Real-Time qPCR

The specificity of the multiplex real-time qPCR assay was determined by utilizing the positive DNA or cDNA of the above-mentioned swine pathogens PCV1, PCV3, PCV4, PRRSV, CSFV, PPV, *Chlamydia suis*, and *Toxoplasma gondii* as detection templates, the plasmid standards as positive controls, and ddH_2_O as a negative control. All templates and ddH_2_O were repeated three times under the optimal reaction conditions for specificity of the multiplex PCR.

The detection limit determines the occurence of false negatives during detection. To analyze the sensitivity of the established multiplex real-time qPCR, the linearization standard plasmids, pEasy-ASFV, pEasy-PCV2, and pEasy-PRV, prepared above were diluted 10-fold serially to a final concentration between 1 × 10^7^ copies/μL and 1 × 10^1^ copies/μL in nuclease-free water. The diluted standard plasmids were used as templates for multiplex qPCR amplification in the optimal reaction conditions.

To analyze the repeatability and reproducibility of the established multiplex real-time qPCR, the linearization standard plasmids, pEasy-ASFV, pEasy-PCV2, and pEasy-PRV, were diluted by a factor of 10 from 10^7^ copies/μL to 10^4^ copies/μL using nuclease-free water. The diluted standard plasmids were used as templates for multiplex real-time qPCR amplification. Using the optimized amplification conditions, each diluted standard plasmid was amplified three times repeatedly and tested every other week. The different concentration groups were compared, and their intra-group and inter-group coefficients of variation were determined to confirm their reproducibility.

### 2.7. Field Specimen Detection

A total of 383 field specimens gathered from four eastern provinces of China were detected using the established real-time qPCR. In brief, all the specimens were resuspended and homogenized in phosphate-buffered saline (PBS). Following the manufacturer’s instructions, the nucleic acid was extracted from a homogenized specimen using a PureLink Viral DNA Mini Kit (ThermoFisher, Waltham, MA, USA). The constructed plasmid and ddH_2_O were employed as positive and negative controls, respectively. The extracted DNA from field specimens was measured by the assay established in this study, and then the epidemiological survey data were analyzed.

## 3. Results

### 3.1. Optimization of the Multiplex Real-Time qPCR Reaction Conditions

After the repeated tests for optimization of primer and probe concentrations, the optimum reaction conditions for multiplex real-time qPCR were obtained as follows: 10 μL 2 × AceQ qPCR Probe Master Mix (Vazyme, Nanjing, China) was used; initial primer concentrations of 10 μM of ASFV-p72qF, ASFV-p72qR, PRV-gEqF, PRV-gEqR, PCV2-CapqF, PCV2-CapqR, β-Actin-qF, and β-Actin-qR were 0.6 μL each; initial probe concentrations of 10 μM of ASFV-probe, PRV-probe, PCV2-probe, and β-Actin-probe were 0.3 μL each; the volume of the DNA template and ddH_2_O was 4 μL; and the total volume of the reaction was 20 μL. The qPCR reaction program was as follows: 95 °C pre-denaturation for 3 min; followed by 40 cycles, which consisted of denaturation at 95 °C for 10 s, annealing at 60 °C for 10 s, and extension at 72 °C for 20 s. Fluorescence channels 1–4 were arranged to amplify the four genes ASFV p72 (FAM), β-Actin (HEX), PCV2 Cap (CY5), and PRV gE (Texas Red), respectively. Finally, all the fluorescence signals were collected by a real-time PCR instrument.

### 3.2. Standard Curves

**Figure 1:**
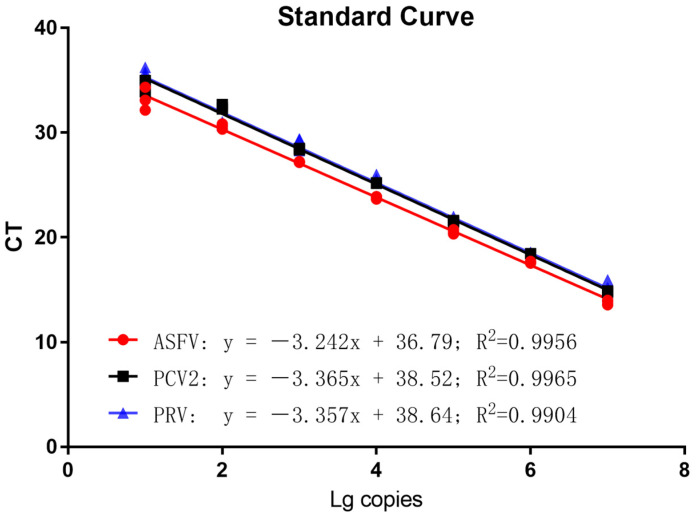
Standard curves of the multiplex real-time qPCR. Establishment of a standard curve for TaqMan probe-based multiplex real-time qPCR. The logarithm of the number of copies is the horizontal axis. The vertical axis represents Ct values. Each point represents the mean value of three duplicates of plasmid that have been diluted. The optimal standard formula for ASFV is y = 3.242x + 36.79, with a correlation coefficient of 0.9956; the optimal standard formula for PCV2 is y = 3.365x + 38.52, with a correlation coefficient of 0.9965; and the optimal standard formula for PRV is y = 3.357x + 38.64, with a correlation coefficient of 0.9904.

Diluted to 10^7^−10^1^ copies/μL in a 10-fold gradient, pEasy-ASFV, pEasy-PCV2, and pEasy-PRV were amplified by multiplex real-time qPCR using the best reaction conditions and procedure. Three standard curves of gene amplification were established using the obtained Ct values and plasmid concentrations. As shown in Figure 1, the correlation coefficients were R^2^ = 0.9956 for ASFV, R^2^ = 0.9965 for PCV2, and R^2^ = 0.9904 for PRV, which proved an excellent correlation coefficient and amplification efficacy. This suggested that the established TaqMan probe-based multiplex real-time qPCR for each virus was reliable and valid.

### 3.3. Specificity of the Multiplex Real-Time qPCR

**Figure 2:**
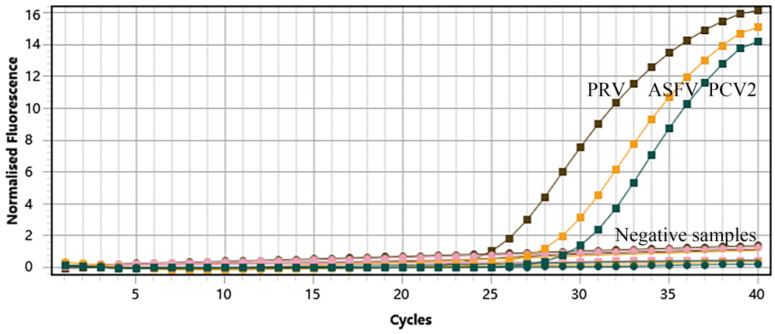
The amplification curves of specificity tests of multiplex real-time qPCR. Only ASFV, PCV2, and PRV exhibited positive fluorescence signals, while other swine pathogens exhibited no fluorescence signals.

There are numerous pathogens in pig farms that might induce reproductive disorders in sows. To evaluate the specificity of the method established in this experiment, we performed specificity tests with prevalent pathogen specimens. The standard plasmids, pEasy-ASFV, pEasy-PCV2, and pEasy-PRV, were tested as positive controls. Positive nucleic acids of JEV, CSFV, PRRSV, PCV1, PCV3, PCV4, PPV, *Chlamydia suis*, and *Toxoplasma gondii* were tested as templates, and ddH_2_O was tested as a negative control. As shown in Figure 2, three S-shaped curves demonstrated that the target genes from the above-constructed plasmids were detected using this assay. However, this method did not detect fluorescence signals of other swine pathogens, resulting in no amplification curves. Moreover, the Ct values obtained by the machine also verified the above results (Table 3). Overall, this indicates that this established method has a high degree of specificity.

### 3.4. Sensitivity of the Multiplex Real-Time qPCR

**Figure 3:**
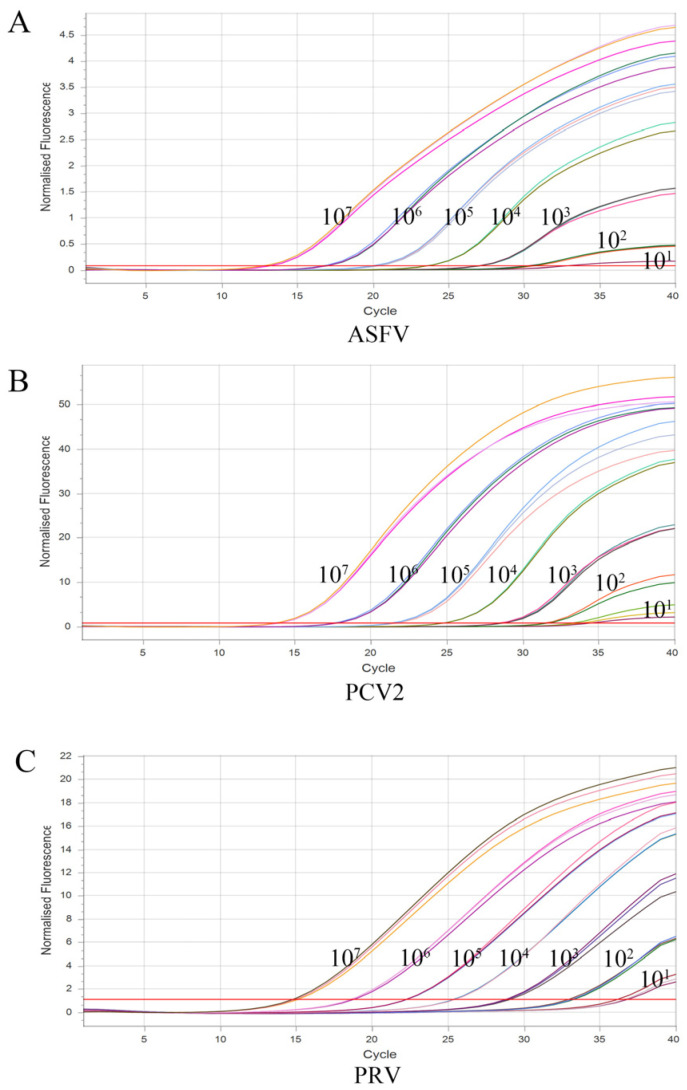
The amplification curves of the sensitivity test of the multiplex real-time qPCR. Multiple real-time qPCR sensitivity test using 10-fold serial dilutions of recombinant plasmids, with three repetitions of each dilution test. (**A**): ASFV sensitivity analysis; (**B**): PCV2 sensitivity analysis; (**C**): PRV sensitivity analysis.

Multiplex real-time qPCR was performed utilizing the most appropriate reaction conditions to amplify the target genes from seven concentrations ranging from 1 × 10^7^ copies/μL to 1 × 10^1^ copies/μL of three constructed plasmids of ASFV, PCV2, and PRV for the sensitivity test. As shown in Figure 3, the results demonstrated that the assay for every viral pathogen was successfully established at the limit of detection at 10^1^ copies/μL.

### 3.5. Repeatability of the Multiplex Real-Time qPCR

The resulting data showed that the variation coefficients (CVs) of Ct values in the intra-group reproducibility tests and inter-group reproducibility tests ranged from 0.39% to 1.20% and from 0.06% to 1.96% (Table 4), respectively, indicating that the method established has good reproducibility.

### 3.6. The Detection of Field Specimens

The established assay was employed to determine the DNA of 383 field specimens collected from East China. As shown in Table 5, the detection data indicated that the single infection rates of ASFV, PCV2, and PRV were 22.45% (86/383), 28.46% (109/383), and 2.87% (11/383), respectively. The co-infection rates of ASFV + PCV2, ASFV + PRV, PCV2 + PRV were 5.22% (20/383), 0.26% (1/383), and 1.83% (7/3383), respectively, while the ASFV + PCV2 + PRV mixed infection rate was 0.26% (1/383). All field specimens were detected using the internal reference primers and probe and showed positive data, suggesting that all the specimens were sampled normally, and no false-positive results were observed.

## 4. Discussion

Sow reproductive disorders still have a serious impact on farms, and the widespread availability of weak strains of ASFV complicates the epidemiology of the disease [36,37]. Given that ASFV, PCV2 and PRV can cause a series of pig diseases, such as reproductive disorders in sows, it is necessary for pig farms to differentiate the three viruses [38]. In addition, missed inspections often occur due to nonstandard sampling. To this end, many established multiplex real-time qPCR methods have been reported to establish the amplification of internal reference genes to avoid false-negative results. Herein, the β-Actin gene was amplified as an internal reference to avoid false-negative results. Therefore, a TaqMan probe-based multiplex real-time qPCR assay was established for ASFV, PCV2, and PRV, which also could detect the internal reference gene β-Actin, thus allowing the simultaneous detection of three pathogens while ensuring the accuracy and reliability of the assay. The tested data confirmed this assay’s high specificity and lack of cross-reactivity with widespread porcine microorganisms. The detection limit of each pathogen can reach 1 × 10^1^ copies/μL, and the intra-group and inter-group variation coefficients are less than 2%. Meanwhile, the assay established has good reproducibility and sensitivity, providing a sensitive and specific tool for the rapid detection of ASFV, PCV2, and PRV in clinical diagnosis.

Virus gene analysis and detection are crucial components of epidemic control and prevention. There are numerous approaches to analyzing genes [39]. PCR is the most commonly used detection and analyzing method in the antigen screening of all kinds of pathogens and is also one of the most important approaches for the early diagnosis of viral infections, including ASFV. An earlier report showed that a qPCR assay was established to detect DNA at 1.0 × 10^1^ copies/μL using the special primers and probe for the conservative region of the ASFV p72 gene. Recently, the wild-type and gene-deleted ASFV strains were detected simultaneously using a multiplex real-time qPCR assay, which tested for ASFV with a detection limit of 32.1 copies/L for the p72 gene and 3.21 copies/L for the I177L, MGF505-2R, and CD2v genes [40]. In addition, multiple reports have shown qPCR assays being used for PRV detection. Cheng et al. [41] developed a qPCR method for detecting PRV gB in saliva specimens and nasal swabs of swine. Zhao et al. [42] established a digital PCR (ddPCR) detection method based on the ORF2 gene of PCV2, and its detection limit could reach 2.5 × 10^1^ copies/μL. Currently, few qPCR methods can concurrently detect these three viruses. Remarkably, the approach developed in this study compensated for the shortcomings of clinical detection. Moreover, its detection limit reached 10^1^ copies/μL for each virus.

Since August 2018, the ASF epidemic has occurred in 31 provinces nationwide, seriously harming the Chinese pig-breeding industry and causing huge economic losses [43]. The ASFV infection rate of 22.45% reflects that there was still a relatively wide spread of ASFV in China from 2020 to 2022. Additionally, 86 ASFV-positive specimens were tested using a pair of special primers for MGF360, and the results showed that there were MGF360 gene-deficient strains among these ASFV-positive specimens. This indicated that the coexistence of wild virus strains and natural variant strains of ASFV has led to a great increase in the infection pressure of pig herds. As a common pathogen in pig farms, a large number of previous studies have reported that PCV2 causes serious infections in many regions of China. Zheng et al. [44] collected 117 field specimens from Henan Province, Central China, from 2015 to 2018 and found that the positivity rate of PCV2 was 62.4%. In 2019, Xu et al. [45] reported that the PCV2-positive rate was 63.16% (84/133), and the PCV2 and PCV4 co-infection-positive rate was 21.05% (28/133) in Henan Province. In South China, Nan et al. [46] collected 573 tissue specimens from 132 pig farms in Northern Guangdong Province during 2016–2021 and investigated the recent prevalence of PCV2 by using PCR. The data showed that 51.38% (297/573) of specimens tested positive for PCV2. Therefore, the recent high positivity rates in some provinces have illustrated the prevalence of PCV2 in China. In this study, we found that the prevalence rate of PCV2 in the field specimens from East China between 2020 and 2022 reached 28.46%, implying an obvious PCV2 infection problem in East China. Because the pig density in East China is significantly lower than that in Henan and Guangdong Provinces, it is understandable that the positivity rate in East China is not as high as those in Central or South China. Interestingly, in a meta-analysis of PCV2 infection rates from 2015 to 2019, the infection rate of PCV2 in East China ranged from 13.5% to 63.5%, which is in line with our findings [47]. Due to the widespread popularity of PRV vaccination, the infection rate of PRV is relatively low. Compared to the prevalence of pathogens in domestic herds in recent years, the low prevalence of infection with PRV suggested that it was not the dominant pathogen causing abortions in sick sows at several pig farms. Therefore, prevention and control should aim to block the spread of ASFV and PCV2 in the field.

## 5. Conclusions

A TaqMan probe-based multiplex real-time qPCR assay was successfully established to detect ASFV, PCV2, and PRV simultaneously, providing a sensitive and specific tool for the rapid detection of swine viral diseases caused by DNA viruses in the field. The detection results also show that there is no obvious prevalence of mixed infection with ASFV and PCV2 or PRV in East China, indicating that the rate of PRV is declining and that PRV will be potentially eradicated in East China. Of course, the detection of ASFV is still the focus of this work, and we should not neglect the prevention and control of PCV2.

## Figures and Tables

**Table 1:** Primers and probes that were employed for this study.

Primers and Probes	Sequences (5′-3′)	Length (Base Pair)	Use
ASFV-p72F	CGAAGGGAATGGATACTGA	563	Amplification of p72
ASFV-p72R	TGTACCCGGCACAAAGA		
ASFV-p72qF	ACGTTCGCTGCGTATCATTT	112	qPCR for detection of p72
ASFV-p72qR	GAGGTATCGGTGGAGGGAAC		
ASFV-probe	FAM- TGCACAAGCCGCACCAAAGCA-TAMRA		
PCV2-CapF	TTACACGGATATTGTATTCCTGGTCG	295	Amplification of Cap
PCV2-CapR	GTGGGCTCCAGTGCTGTTATTCTA		
PCV2-CapQF	AGTCTCAGCCACAGCTGATT	128	qPCR for detection of Cap
PCV2-CapQR	TCCTCCCGCCATACCAT		
PCV2-probe	Cy5-AGCCCTTCTCCTACCACTCCCGCT-BHQ2		
PRV-gEF	GCGGCTGTTTGTGCTG	413	Amplification of gE
PRV-gER	CATAGTTGGGTCCATTCGT		
PRV-gEqF	GCTCCTTCGTGATGACGTG	131	qPCR for detection of gE
PRV-gEqR	GTACACCGGAGAGAGCATGT		
PRV-Probe	Texas Red-CTGCGTGCTGTGCTCCCGGC-BHQ2		
β-ActinqF	CCCTGGAGAAGAGCTACGAG	175	qPCR for detection of β-Actin [34]
β-ActinqR	AGGTCCTTCCTGATGTCCAC		
β-Actin-probe	HEX-CGGCAACGAGCGCTTCCGGT-BHQ1		

**Table 2:** The information of field specimens detected in this study.

Province	Number	Characteristic	Specimen Type
Jiangsu	144	Sows	Lymph nodes, sera, and/or blood
		Aborted piglets	Lymph nodes and/or placentas
Anhui	107	Sows	Lymph nodes, sera, and/or blood
		Aborted piglets	Lymph nodes and/or placentas
Zhejiang	72	Sows	Lymph nodes, sera, and/or blood
Shandong	60	Sows	Lymph nodes, sera, and/or blood

**Table 3:** The Ct values of the specificity tests of the multiplex real-time qPCR.

Swine Pathogens	Ct Value ^a^			
	FAM	HEX	Cy5	Texas Red
ASFV	25.22 ± 0.19	-	-	-
PCV2	-	-	27.25 ± 0.21	-
PRV	-	-	-	24.73 ± 0.24
PCV1	-	-	-	-
PCV3	-	-	-	-
PCV4	-	-	-	-
CSFV	-	-	-	-
JEV	-	-	-	-
PPV	-	-	-	-
PRRSV	-	-	-	-
*Chlamydia suis*	-	-	-	-
*Toxoplasma gondii*	-	-	-	-

^a^ Data are shown as means ± SD from three independent experiments.

**Table 4:** Repeatability analysis of the multiplex real-time qPCR.

Plasmid	Concentration (Copies/μL)	Intra-Assay			Inter-Assay		
		Mean	S.D.	CV (%)	Mean	S.D.	CV (%)
pEasy-ASFV	10^7^	15.36	0.132	0.86	15.21	0.058	0.39
	10^6^	18.19	0.078	0.43	18.12	0.012	0.06
	10^5^	21.44	0.083	0.39	21.57	0.073	0.34
	10^4^	25.47	0.276	1.08	25.05	0.493	1.96
pEasy-PCV2	10^7^	15.53	0.104	0.67	15.47	0.048	0.31
	10^6^	18.8	0.139	0.74	18.88	0.063	0.33
	10^5^	20.85	0.237	1.14	21.47	0.380	1.73
	10^4^	24.76	0.297	1.20	25.04	0.203	0.81
pEasy-PRV	10^7^	15.53	0.104	0.67	15.49	0.027	0.18
	10^6^	18.80	0.139	0.74	18.77	0.134	0.71
	10^5^	20.85	0.237	1.14	20.81	0.127	0.61
	10^4^	24.76	0.297	1.20	24.70	0.134	0.54

**Table 5:** Detection of field specimens by multiplex real-time qPCR.

Virus	Positive Specimens ^a^	Infection Rate (%)
ASFV	86	22.45
PCV2	109	28.46
PRV	11	2.87
ASFV + PCV2	20	5.22
ASFV + PRV	1	0.26
PRV + PCV2	7	1.83
ASFV + PCV2 + PRV	1	0.26
β-Actin	383	100
Total	383	/

^a^ These data represent three independent experiments.

## Data Availability

Not applicable.
